# Physical frailty, genetic predisposition, and incident dementia: a large prospective cohort study

**DOI:** 10.1038/s41398-024-02927-7

**Published:** 2024-05-27

**Authors:** Pei-Yang Gao, Ling-Zhi Ma, Xue-Jie Wang, Bang-Sheng Wu, Yi-Ming Huang, Zhi-Bo Wang, Yan Fu, Ya-Nan Ou, Jian-Feng Feng, Wei Cheng, Lan Tan, Jin-Tai Yu

**Affiliations:** 1 Department of Neurology, Qingdao Municipal Hospital, Qingdao University, Qingdao, China; 2 Department of Neurology and National Center for Neurological Disorders, Huashan Hospital, State Key Laboratory of Medical Neurobiology and MOE Frontiers Center for Brain Science, Shanghai Medical College, Fudan University, Shanghai, China; 3 Innovation Center for Neurological Disorders and Department of Neurology, Xuanwu Hospital, Capital Medical University, National Center for Neurological Disorders, Beijing, China; 4 Institute of Science and Technology for Brain-Inspired Intelligence, Fudan University, Shanghai, China; 5 Key Laboratory of Computational Neuroscience and Brain-Inspired Intelligence (Fudan University), Ministry of Education, Shanghai, China; 6 Fudan ISTBI—ZJNU Algorithm Centre for Brain-Inspired Intelligence, Zhejiang Normal University, Jinhua, China; 7 MOE Frontiers Center for Brain Science, Fudan University, Shanghai, China; 8 Zhangjiang Fudan International Innovation Center, Shanghai, China

**Keywords:** Diseases, Neuroscience

## Abstract

Physical frailty and genetic factors are both risk factors for increased dementia; nevertheless, the joint effect remains unclear. This study aimed to investigated the long-term relationship between physical frailty, genetic risk, and dementia incidence. A total of 274,194 participants from the UK Biobank were included. We applied Cox proportional hazards regression models to estimate the association between physical frailty and genetic and dementia risks. Among the participants (146,574 females [53.45%]; mean age, 57.24 years), 3,353 (1.22%) new-onset dementia events were recorded. Compared to non-frailty, the hazard ratio (HR) for dementia incidence in prefrailty and frailty was 1.396 (95% confidence interval [CI], 1.294–1.506, *P* < 0.001) and 2.304 (95% CI, 2.030–2.616, *P* < 0.001), respectively. Compared to non-frailty and low polygenic risk score (PRS), the HR for dementia risk was 3.908 (95% CI, 3.051–5.006, *P* < 0.001) for frailty and high PRS. Furthermore, among the participants, slow walking speed (HR, 1.817; 95% CI, 1.640–2.014, *P* < 0.001), low physical activity (HR, 1.719; 95% CI, 1.545–1.912, *P* < 0.001), exhaustion (HR, 1.670; 95% CI, 1.502–1.856, *P* < 0.001), low grip strength (HR, 1.606; 95% CI, 1.479–1.744, *P* < 0.001), and weight loss (HR, 1.464; 95% CI, 1.328–1.615, *P* < 0.001) were independently associated with dementia risk compared to non-frailty. Particularly, precise modulation for different dementia genetic risk populations can also be identified due to differences in dementia risk resulting from the constitutive pattern of frailty in different genetic risk populations. In conclusion, both physical frailty and high genetic risk are significantly associated with higher dementia risk. Early intervention to modify frailty is beneficial for achieving primary and precise prevention of dementia, especially in those at high genetic risk.

## Introduction

Dementia is a chronic progressive syndrome characterized by a more severe decline in cognitive function compared to the normal aging process [1]. With its associated increased morbidity and mortality, dementia imposes a significant financial burden on countries, societies, and families [2]. However, dementia treatment is elusive because of the insufficient efficacy of existing medications; therefore, prevention strategies is a viable and effective approach to decreasing the global dementia incident [3, 4]. Among these, modifiable risk factors for the target population, including better physical function and regular daily activities, are critical for primary and precise prevention and can benefit in reducing the risk of incident dementia [5–8].

Physical frailty is a multidimensional description of the body’s state, and is accompanied by a multisystemic decline in function and increased vulnerability to stressors. It is a consequence of the normal aging process and is both dynamic and modifiable [9–11]. Thus, physical frailty is an important modifiable factor in the risk of dementia [12–16]. The evaluation of frailty typically involves two methods. The first, proposed by Fried et al., includes five components: weight loss, exhaustion, low grip strength, slow walking speed, and low physical activity [12]. The second method, introduced by Rockwood and colleagues, defines a Frailty Index based on 49 clinical indicators [17]. However, the practical application favors the methodology introduced by Fried et al., due to the ease of observation of its five evaluative criteria, which holds more significant clinical relevance, leading to its more widespread adoption [18, 19].

Meanwhile, genetic risk has been acknowledged as an independent, non-modifiable risk factor for dementia [20]. The polygenic risk score (PRS) is a quantitative tool for assessing the genetic risk of an individual and is widely used in clinical practice and epidemiological investigations [6, 21, 22]. Early primary and precise prevention strategies are necessary for individuals at higher dementia genetic risk, and earlier modification of physical frailty status may allow individuals to reduce the risk of developing dementia [23]. However, limited evidence exists on the association of physical frailty, genetic predisposition, and their joint effect with dementia risk.

Therefore, the main objective of this study was to explore the longitudinal associations among physical frailty, genetic risk, and the risk of incident dementia using the UK Biobank. Physical frailty phenotype (categorized as non-frailty, pre-frailty, and frailty) and dementia PRS (categorized as low, intermediate, and high) were used for the main analysis to explore: (1) the association of physical frailty phenotype and genetic risk with incident dementia; (2) the effect of physical frailty phenotype on the expression of genetic risk of dementia; and (3) the relationship between the five components of physical frailty and dementia risk among all the participants and different PRS classifications for precise prevention.

## Materials and methods

### Study population

The UK Biobank is a large population-based biological and medical database and a research project. It consists of detailed genetic and bio-information from over 500,000 UK individuals who were recruited at 22 centers across the UK between 2006 and 2010 [24]. Electronic health data were collected using touchscreen questionnaires, physical examinations, sampling testing, and genotyping. Authorization and electronic signatures were obtained from each participant (https://www.ukbiobank.ac.uk/).

This study included 502,408 individuals from the UK Biobank. We excluded participants who were younger than 40 years (*n* = 7), were diagnosed with dementia at baseline (*n* = 975), died within 2 years from baseline (*n* = 2504), had unavailable follow-up information (*n* = 69,731), and without dementia PRS (*n* = 126,635) or physical frailty data (*n* = 28,362). Finally, 274,194 UK Biobank participants were included in the main analysis (Supplementary eFig. 1).

### Physical frailty assessment

Physical frailty was assessed according to the classification scheme proposed by Fried et al., which includes five components: weight loss (Field ID: 2306), exhaustion (Field ID: 2080), low grip strength (Field ID: 46, 47), slow walking speed (Field ID: 924), and low physical activity (Field ID: 6164) (Further details are provided in the Supplementary eTable 1) [12]. Participants who met more than three evaluation criteria and one or two evaluation criteria were considered to be in the frailty and prefrailty categories, respectively. Only those who did not meet any of the criteria were considered to be in the non-frailty category.

### Polygenic risk score

The UK Biobank project has outlined the genotyping, imputation, and quality control of genetic data (http://www.ukbiobank.ac.uk/scientists-3/genetic-data/). Using the PRSice software (www.PRSice.info), we generated numerous genetic risks according to the International Genomics of Alzheimer’s Disease Project (IGAP) for a genome-wide association meta-analysis of people of European ancestry with dementia scores [25, 26]. Hence, genetic investigations have been limited to Caucasians (British, Irish, or other whites). Single nucleotide polymorphisms (SNPs) with call rates of 95%, minor allele frequencies of 0.1%, and *P* < 1 × 10^–10^ departures from Hardy–Weinberg equilibrium were excluded. In this study, *P*-informed aggregation with R^2^ = 0.01 truncation in a 250-kb window was used. Higher PRS scores indicated a greater genetic predisposition to Alzheimer’s disease for all SNPs related to the condition. As in previous studies, we used the PRS, which combines multiple risk alleles for Alzheimer’s disease, to predict the occurrence of all-cause dementia and provide a quantitative measure of genetic dementia risk [6, 27, 28]. Based on previously studies, the participants were further categorized into three groups according to their dementia PRS, namely low (lowest quintile), intermediate (quintiles 2–4), and high (highest quintile) [6, 29].

### Dementia diagnosis

Hospital inpatient records containing admission and diagnostic data from the Welsh Patient Episode Database, Scottish Morbidity Record data, and English Hospital Episode Statistics were analyzed to identify the presence of dementia. Additionally, the occurrence of dementia was revealed via cross-referencing death records from the Information and Statistics Division for Scotland and the National Health Service Digital for England and Wales. The codes applied in the dementia diagnoses are listed in Supplement eTable2 [30, 31]. Follow-up information and participant survival time were calculated from the date of the first evaluation, baseline date, to the date of first occurrence, including dementia diagnosis, termination to follow-up, death, or update date of interconnections.

### Covariates

Baseline information including age (Field ID: 21022); sex (categorized as female and male; Field ID: 31); education levels, categorized as higher (college or university degree) and lower (other degree) level (Field ID: 6138), and assessment centers (Field ID: 54) were collected. Moreover, smoking status (Field ID: 20116) and alcohol intake (Field ID: 20117), categorized as previous use, current use, or never used, were included as covariates. Body mass index (BMI; Field ID: 23104) was determined using impedance measurements. The Townsend deprivation index (TDI; Field ID: 22189), an area-based measure in which participants are assigned a relative score based on the zip code of their residence and is used to distinguish between participants’ residential areas, was also included as a covariate [32]. 27 Furthermore, the number of long-term morbidities (categorized as 0, 1, 2, 3, 4, and ≥5; Field ID: 20002), including 42 major chronic conditions was another covariate considered, which was assessed by experienced professionals through a verbal interview at baseline (Supplementary eTable 3). Finally, a genotyping array (Field ID: 22000), dementia PRS, and the first 40 principal components of ancestry were used as covariates.

### Statistical analysis

Continuous variables, presented as mean and standard deviation (SD), were used to describe the basic characteristics across the three different physical frailty phenotypes. Categorical variables are reported as numbers and percentages.

To examine the association between physical frailty, genetic predisposition, and incident dementia, Cox proportional hazard regression models were performed using the ‘survival’ package in R software, with the length of the follow-up period as the basis for measuring time; hazard ratios (HRs) and 95% confidence intervals (CIs) were reported. To verify the underlying assumptions of the Cox model, we employed the log-rank test and Kaplan-Meier survival curves for validation [33, 34]. The results confirmed that they are consistent with the assumptions. The related outcomes are presented in the Supplementary eFigs. 3–5 (log-rank *P* < 0.05). We first divided the population into three groups, non-frailty, prefrailty, and frailty, based on the frailty phenotype, and non-frailty was used as a reference to calculate the HRs (95% CI), as well as, incident cases per 100,000 person-years. To explore the combined effect of frailty phenotype and genetic predisposition on the risk of incident dementia, the recruited participants were categorized into nine groups, and HRs (95% CI) were calculated for each group using Cox proportional hazard regression models, with non-frailty and low PRS as references. Stratified analyses based on the PRS were performed. Moreover, to investigate the necessity of specific dementia prevention strategies for different populations, the relationship between the five components of frailty assessment and risk of incident dementia was evaluated separately for all participants and different PRS categories. Finally, subgroup analyses, including sex, age, TDI, and number of long-term morbidities, were performed to examine the correlation between frailty and the risk of incident dementia.

The evaluation of the sample size using G*Power (version 3.1) demonstrated a power exceeding 99.99% at a significance level of 5% (two-sided), indicating that the sample size for this study is adequately powered. We reported conventional and significant two-sided *P*-value thresholds of 0.05 and 95% CI. The R Studio software (version 4.2.1) was used for all statistical analyses and data visualization.

## Results

### Baseline characteristics

Table 1 presents the baseline demographics and features grouped by physical frailty phenotype. After a mean follow-up of 8.70 (SD 2.52) years (2,385,177 person-years), a total of 274,194 participants (mean age at baseline, 57.24 [SD 7.91] years and 146,574 [53.45%] females), including 150,913 (55.04%) non-frailty, 111,409 (40.63%) prefrailty, and 11,872 (4.33%) frailty individuals were enrolled and 3,353 dementia cases were recorded. Compared to non-frailty, pre-frailty and frailty were older, predominantly female, a higher TDI, and more chronic diseases.

**Table 1:** Baseline Characteristics of Participants in the UK Biobank.

	Frailty phenotype			
Baseline characteristics	Non-frailty	Prefrailty	Frailty	*P* value
Participants No.	150,913	111,409	11,872	
Age, mean (SD), years	56.79 (7.95)	57.68 (7.88)	58.71 (7.35)	<0.001
Higher education levels, No. (%)	53,501 (35.45)	29,804 (26.75)	1,664 (14.02)	<0.001
Sex, No. (%)				<0.001
Female	75,404 (49.97)	63,673 (57.15)	7,497 (63.15)	
Male	75,509 (50.03)	47,736 (42.85)	4,375 (36.85)	
Smoking, No. (%)				<0.001
Never	84,646 (56.23)	57,791 (52.06)	5,016 (42.56)	
Previous	53,125 (35.29)	40,670 (36.63)	4,429 (37.58)	
Current	12,763 (8.48)	12,556 (11.31)	2,340 (19.86)	
Alcohol, No. (%)				<0.001
Never	3,551 (2.35)	4,034 (3.62)	802 (6.77)	
Previous	3,429 (2.27)	4,665 (4.19)	1,341 (11.32)	
Current	14,3881 (95.37)	102,608 (92.18)	9,701 (81.91)	
Body mass index, mean (SD), kg/m^2^	26.60 (4.08)	28.30 (5.03)	31.30 (6.69)	<0.001
Townsend deprivation index, mean (SD)	−1.92 (2.72)	−1.31 (3.04)	0.12 (3.47)	<0.001
Number of morbidities, No. (%)				<0.001
0	63,318 (41.96)	31,493 (28.27)	1,006 (8.47)	
1	50,355 (33.37)	35,427 (31.80)	2,202 (18.55)	
2	24,085 (15.96)	23,700 (21.27)	2,673 (22.52)	
3	8,941 (5.92)	12,056 (10.82)	2,400 (20.22)	
4	2,927 (1.94)	5,268 (4.73)	1,639 (13.81)	
5 or more	1,287 (0.85)	3,465 (3.11)	1,952 (16.44)	
Genetic risk category, No. (%)				0.940
Low	30,155 (19.98)	22,320 (20.03)	2,364 (19.91)	
Intermediate	90,646 (60.07)	66,755 (59.92)	7,116 (59.94)	
High	30,112 (19.95)	22,334 (20.05)	2,392 (20.15)	

The continuous variables and categorical variables were indicated as mean (SD) and number (percentage), respectively.

### Associations between physical frailty, genetic risk, and risk of incident dementia

Dementia risk increased steadily across the three physical frailty phenotypes. The incidence of dementia cases per 100,000 person-years was 101.10, 167.74, and 369.31 for non-frailty, prefrailty, and frailty, respectively (Table 2). In addition, compared to non-frailty, after adjusting for covariates, prefrailty and frailty had a significantly elevated dementia risk; the HR for prefrailty and frailty was 1.396 (95% CI, 1.294–1.506; *P* < 0.001) and 2.304 (95% CI, 2.030–2.616; *P* < 0.001), respectively.

**Table 2:** Association between physical frailty and risk of incident dementia.

			Model 1			Model 2		
Frailty phenotype	Cases/ person-years	Incident cases per 100,000 person-years	HR (95% CI)	*P*	*P* for trend	HR (95% CI)	*P*	*P* for trend
Non-frailty	1,314/1,299,758	101.10	1 [Reference]			1 [Reference]		
Prefrailty	1,639/977,109	167.74	1.452 (1.349–1.563)	<0.001	<0.001	1.396 (1.294–1.506)	<0.001	<0.001
Frailty	400/108,310	369.31	2.724 (2.426–3.058)	<0.001		2.304 (2.030–2.616)	<0.001	

Model 1, adjusted for age, sex, education levels, Townsend deprivation index, and assessment centers.

Model 2, adjusted for model 1 plus alcohol intake, smoking status, body mass index, the number of long-term morbidities, polygenic risk score, genotyping array, and the first 40 principal components of ancestry.

**Fig. 1:**
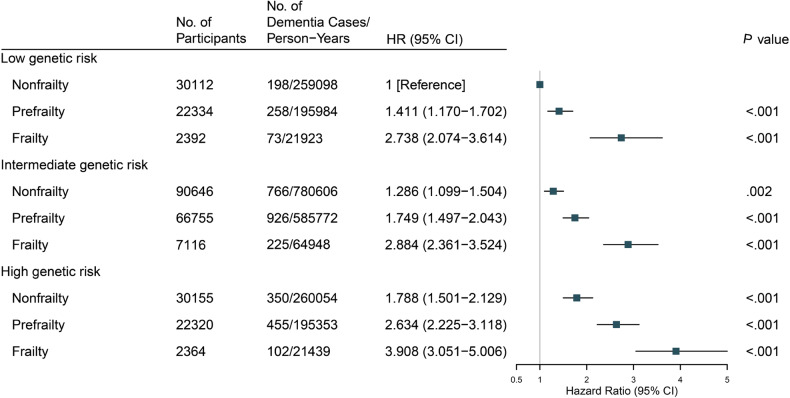
Risk of incident dementia according to polygenic risk score and physical frailty phenotype. Model adjusted for age, sex, education levels, Townsend deprivation index, assessment centers, alcohol intake, smoking status, body mass index, the number of long-term morbidities, genotyping array, and the first 40 principal components of ancestry.

The monotonically increasing risk of incident dementia across the PRS categories was evaluated (Supplementary eTable 4). We further found a monotonic association when genetic risk and frailty phenotypes were combined, indicating that individuals with increasing frailty and PRS had a higher risk of incident dementia (Fig. 1). Compared with non-frail participants with low PRS, frail participants with high PRS were significantly associated with a higher risk of incident dementia (HR, 3.908; 95% CI, 3.051–5.006; *P* < 0.001). Notably, the HR for dementia risk in non- frailty individuals with high PRS was remarkably lower than that in frail individuals with low or intermediate PRS (1.788 [95% CI, 1.501–2.129; *P* < 0.001] vs. 2.884 [95% CI, 2.361–3.524; *P* < 0.001] vs. 2.738 [95% CI, 2.074–3.614; *P* < 0.001], respectively) and was similar to the risk in prefrail individuals with intermediate PRS (HR, 1.749; 95% CI, 1.497–2.043; *P* < 0.001), suggesting the enormous importance of improving physical frailty for dementia prevention.

### Associations between five components and risk of incident dementia

**Fig. 2:**
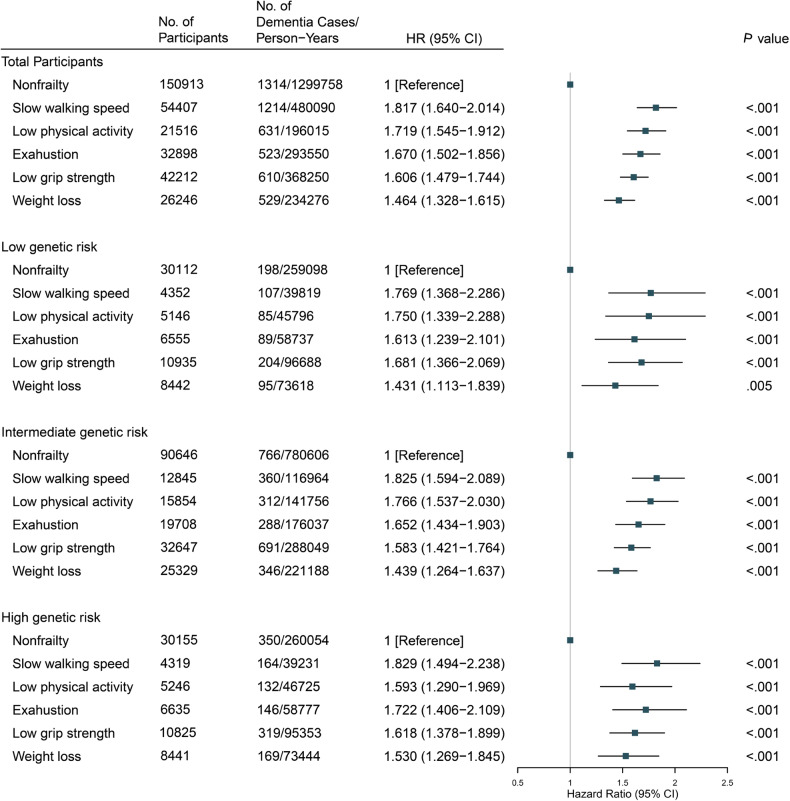
Risk of incident dementia according to five components of physical frailty within each genetic risk category. Model adjusted for age, sex, education levels, Townsend deprivation index, assessment centers, alcohol intake, smoking status, body mass index, the number of long-term morbidities, genotyping array and the first 40 principal components of ancestry.

The association of the five components comprising the evaluation of physical frailty with dementia risk was also separately investigated among all participants and different PRS categories (Fig. 2). In total participants, compared to non-frailty, slow walking speed (HR, 1.817; 95% CI, 1.640–2.014, *P* < 0.001), low physical activity (HR, 1.719; 95% CI, 1.545–1.912, *P* < 0.001), exhaustion (HR, 1.670; 95% CI, 1.502–1.856, *P* < 0.001), low grip strength (HR, 1.606; 95% CI, 1.479–1.744, *P* < 0.001), and weight loss (HR, 1.464; 95% CI, 1.328–1.615, *P* < 0.001) were significantly associated with dementia risk. Notably, the ranking of HR values for five components was different in the low- and high-risk populations, illustrating the need for precise setting of dementia prevention strategies for different genetic risk populations.

### Subgroups analysis

Compared with frail individuals, non-frail individuals had a significantly decreased risk of incident dementia; the HR for the low, intermediate, and high PRS category was 0.389 (95% CI, 0.285–0.530; *P* < 0.001), 0.436 (95% CI, 0.369–0.516; *P* < 0.001), and 0.469 (95% CI, 0.365–0.603; *P* < 0.001) (Table 3). Furthermore, the stratified analyses (Supplementary eTable 5) showed a more pronounced association between physical prefrailty and frailty and dementia risk were identified in the age (*P*_interaction_ < 0.001 for pre-frailty; *P*_interaction_ < 0.001 for frailty) and number of morbidities (*P*_interaction_ < 0.037 for pre-frailty; *P*_interaction_ < 0.022 for frailty) subgroups.

**Table 3:** Association between physical frailty and risk of incident dementia within genetic risk classification.

	PRS, HR (95% CI), *P* value					
Frailty phenotype	Low		Intermediate		High	
Frailty	1 [Reference]		1 [Reference]		1 [Reference]	
Prefrailty	0.543 (0.410–0.720)	<0.001	0.595 (0.509–0.695)	<0.001	0.684 (0.543–0.862)	0.001
Non-frailty	0.389 (0.285–0.530)	<0.001	0.436 (0.369–0.516)	<0.001	0.469 (0.365–0.603)	<0.001

Model adjusted for age, sex, education levels, Townsend deprivation index, education levels, assessment centers, alcohol intake, smoking status, body mass index, the number of long-term morbidities, polygenic risk score, genotyping array, and the first 40 principal components of ancestry.

## Discussion

We investigated 274,194 participants and found that prefrailty and frailty were significantly associated with a higher risk of incident dementia compared to non-frailty. We also identified the joint effect of physical frailty phenotype and genetic risk; frail participants with higher PRS had a higher risk of incident dementia than non-frail participants with low PRS. Furthermore, a strong correlation between five components of physical frailty and dementia risk was found; and a differential risk of dementia in populations with different dementia genetic risks due to frailty composition patterns was also identified.

With the recent increase in the aging population, the prevalence of frailty has increased rapidly [9]. A systematic meta-analysis found that the prevalence of physical frailty in 62 countries and 1,755,497 participants was 15% for females and 11% for males, which implies a significant hazard for public health [35]. Meanwhile, a microsimulation modeling study in Japan reported that both frailty and dementia will impose serious medical, economic, and social burdens in the future [36]. Previous cohort studies have shown a significant association between frailty and dementia and that both of these have common risk factors, such as socio-demographic, long-term morbidities, and lifestyle factors [16, 37–39]. Moreover, a cohort study of 165 older adults from the Rush Memory and Aging Project found that a state of physical frailty before death was significantly associated with AD pathology levels examined postmortem, indicating a possibility of a common pathogenesis between physical frailty and AD pathology [13]. This also provides an exploration of the correlation between frailty and dementia onset in terms of potential mechanisms, although more evidence from experimental, animal, or cellular studies is required.

The main results that a higher risk of developing dementia was significantly associated with poorer physical frailty and higher dementia-related PRS were found. Specifically, a noticeably reduced risk of dementia in non-frail populations with high PRS compared to frail populations with low or intermediate PRS were also identified. This indicates that the physical frailty phenotype may be a factor attenuating the genetic risk of dementia. Unlike genetic risk, physical frailty may be a modifiable risk factor, and the manifestation of physical frailty can be reversed by changing lifestyle or physical exercise habits. Therefore, delaying the development of dementia by modifying physical frailty status can be offered as an option [40]. Moreover, our findings suggest that even with a higher PRS of dementia, public strategies to improve physical capacity may help reduce the burden of physical frailty, thereby reducing the risk of dementia due to frailty [14, 23].

The five components of physical frailty were also strongly associated with an increased risk of incident dementia. Kuo et al. proposed a significant association of low grip strength and slow walking speed with an increased risk of incident dementia [41]. This may be because in the central nervous system, the implementation of these two factors is achieved in areas normally located under the frontal and parietal cortex and in the visuomotor and executive areas of the cortex, which are also associated with the onset of dementia [42–44]. For exhaustion, Sulkava et al. reported an HRs of 1.12 (95% CI, 1.00–1.25) in Finland and S. Islamoska et al. identified an incidence rate ratio of 1.024 (95% CI, 1.004–1.043) in Copenhagen [45, 46]. Weight loss was also identified as an important risk factor of dementia. Previous studies have presented a 34% (95% CI, 29–38%) higher risk for underweight (BMI < 20 kg/m^2^) individuals and 29% (95% CI, 23–34%) decreased risk per 5-kg/m^2^ increase in BMI, which is consistent with our findings [47, 48]. Furthermore, the elderly population often exhibit low levels of physical activity due to declining function and muscle atrophy, which have also been recognized to be associated with dementia incidence, subjective memory decline, and developing all-cause mortality [49–51]. The underlying mechanism for its contribution to the development of dementia is thought to be that exercise modulates the onset of inflammation, synthesis and release of neurotrophins, and alteration of cerebral blood flow [52]. Interestingly, the HR value for slow walking speed demonstrated the highest magnitude of association across the different genetic risk populations. Moreover, among the PRS groups examined, exhaustion exhibited the second-highest HR value for all groups, except the high-genetic-risk population that ranked fourth highest. This suggests that different strategies for the prevention of dementia should be targeted at individuals with different genetic risks, which contributes to successful precise prevention.

Our study has several strengths. The UK Biobank was used as a large-scale, prospective, and long-term follow-up cohort with data on extensive possible covariates, health conditions, and genetic information, which could be adjusted for multiple covariates and stratified into different subgroups in the analysis. However, this study had several limitations. First, compared to a previously reported database, the UK Biobank had a lower prevalence of dementia because the individuals were younger, healthier, and more well-educated. In addition, dementia events were identified only using hospital records and death registers; therefore, missing dementia data were unavoidable. Second, the UK biobank does not include data on recognized markers of dementia pathology (measured using cerebrospinal fluid AD biomarkers, such as amyloid-β and tau, or PET imaging), and does not have results for global cognitive measures (including the Mini-Mental State Examination [MMSE] and the Montreal Cognitive Assessment [MOCA]). Third, we did not use data from all UK Biobank participants because many of them lacked data on frailty assessments or confounders; therefore, they were excluded from this study, which may have introduced bias. Fourth, all four of the five factors assessed for frailty, except low grip strength, were self-reported; therefore, reporting bias was inevitable. However, a previous study by Theou et al. identified similar features of frailty regardless of self-report or test-based measures [53]. Fifth, the majority of participants included in this study were British or European; therefore, the results may not be applicable to other populations, including African, Asian, and Latin American populations. Therefore, further research in other ethnic groups is necessary. Finally, although our analysis was corrected for underlying confounders, this was not adequately evaluated, which could have resulted in bias.

In conclusion, both physical frailty and high genetic risk are significantly associated with an increased risk of dementia. Furthermore, the joint effect of physical frailty phenotypes and dementia genetic predisposition was also identified, suggesting that physical frailty and high genetic risk are significantly associated with higher dementia risk than non-frailty and low genetic risk. Since frailty is a modifiable risk factor, early interventions in frail populations can be recommended to achieve primary prevention of dementia incidence; in particular, different patterns of frailty composition in those at different genetic risks should be considered for precise prevention.

### Supplementary information

Supplementary Material

## Data Availability

All data generated or analyzed during this study are included in this publication and/or are available from the corresponding author on reasonable request.

## Code availability

All software used in this study is publicly available. The code used in this study can be accessed at https://github.com/peiyanggao/UKB_Frailty_Genetic_Dementia/.

## References

[1] Livingston G, Huntley J, Sommerlad A, Ames D, Ballard C, Banerjee S (2020). Dementia prevention, intervention, and care: 2020 report of the Lancet Commission. Lancet.

[2] Barnett K, Mercer SW, Norbury M, Watt G, Wyke S, Guthrie B (2012). Epidemiology of multimorbidity and implications for health care, research, and medical education: a cross-sectional study. Lancet.

[3] Zhang Y, Chen SD, Deng YT, You J, He XY, Wu XR, et al. Identifying modifiable factors and their joint effect on dementia risk in the UK Biobank. Nat Hum Behav. 2023. 10.1038/s41562-023-01585-x 37024724

[4] Sabia S, Singh-Manoux A (2023). Healthy lifestyles for dementia prevention. BMJ.

[5] Jia J, Zhao T, Liu Z, Liang Y, Li F, Li Y (2023). Association between healthy lifestyle and memory decline in older adults: 10 year, population based, prospective cohort study. BMJ.

[6] Lourida I, Hannon E, Littlejohns TJ, Langa KM, Hypponen E, Kuzma E (2019). Association of lifestyle and genetic risk with incidence of dementia. JAMA.

[7] Verghese J, Lipton RB, Katz MJ, Hall CB, Derby CA, Kuslansky G (2003). Leisure activities and the risk of dementia in the elderly. N Engl J Med.

[8] Yamazaki Y, Zhao N, Caulfield TR, Liu CC, Bu G (2019). Apolipoprotein E and Alzheimer disease: pathobiology and targeting strategies. Nat Rev Neurol.

[9] Hoogendijk EO, Afilalo J, Ensrud KE, Kowal P, Onder G, Fried LP (2019). Frailty: implications for clinical practice and public health. Lancet.

[10] Markle-Reid M, Browne G (2003). Conceptualizations of frailty in relation to older adults. J Adv Nurs.

[11] Dent E, Martin FC, Bergman H, Woo J, Romero-Ortuno R, Walston JD (2019). Management of frailty: opportunities, challenges, and future directions. Lancet.

[12] Fried LP, Tangen CM, Walston J, Newman AB, Hirsch C, Gottdiener J (2001). Frailty in older adults: evidence for a phenotype. J Gerontol A Biol Sci Med Sci.

[13] Buchman AS, Schneider JA, Leurgans S, Bennett DA (2008). Physical frailty in older persons is associated with Alzheimer disease pathology. Neurology.

[14] Petermann-Rocha F, Lyall DM, Gray SR, Esteban-Cornejo I, Quinn TJ, Ho FK (2020). Associations between physical frailty and dementia incidence: a prospective study from UK Biobank. Lancet Healthy Longev.

[15] Wallace LMK, Theou O, Godin J, Andrew MK, Bennett DA, Rockwood K (2019). Investigation of frailty as a moderator of the relationship between neuropathology and dementia in Alzheimer’s disease: a cross-sectional analysis of data from the rush memory and aging Project. Lancet Neurol.

[16] Solfrizzi V, Scafato E, Lozupone M, Seripa D, Schilardi A, Custodero C (2019). Biopsychosocial frailty and the risk of incident dementia: the Italian longitudinal study on aging. Alzheimers Dement.

[17] Clegg A, Young J, Iliffe S, Rikkert MO, Rockwood K (2013). Frailty in elderly people. Lancet.

[18] Hanlon P, Nicholl BI, Jani BD, Lee D, McQueenie R, Mair FS (2018). Frailty and pre-frailty in middle-aged and older adults and its association with multimorbidity and mortality: a prospective analysis of 493 737 UK Biobank participants. Lancet Public Health.

[19] Chin M, Kendzerska T, Inoue J, Aw M, Mardiros L, Pease C (2023). Comparing the hospital frailty risk score and the clinical frailty scale among older adults with chronic obstructive pulmonary disease exacerbation. JAMA Netw Open.

[20] Loy CT, Schofield PR, Turner AM, Kwok JB (2014). Genetics of dementia. Lancet.

[21] Polygenic Risk Score Task Force of the International Common Disease A. (2021). Responsible use of polygenic risk scores in the clinic: potential benefits, risks and gaps. Nat Med.

[22] Wand H, Lambert SA, Tamburro C, Iacocca MA, O’Sullivan JW, Sillari C (2021). Improving reporting standards for polygenic scores in risk prediction studies. Nature.

[23] Ward DD, Ranson JM, Wallace LMK, Llewellyn DJ, Rockwood K (2022). Frailty, lifestyle, genetics and dementia risk. J Neurol Neurosurg Psychiatry.

[24] Sudlow C, Gallacher J, Allen N, Beral V, Burton P, Danesh J (2015). UK biobank: an open access resource for identifying the causes of a wide range of complex diseases of middle and old age. PLoS Med.

[25] Andrews SJ, Fulton-Howard B, Goate A (2020). Interpretation of risk loci from genome-wide association studies of Alzheimer’s disease. Lancet Neurol.

[26] Lambert JC, Ibrahim-Verbaas CA, Harold D, Naj AC, Sims R, Bellenguez C (2013). Meta-analysis of 74,046 individuals identifies 11 new susceptibility loci for Alzheimer’s disease. Nat Genet.

[27] Adams HH, de Bruijn RF, Hofman A, Uitterlinden AG, van Duijn CM, Vernooij MW (2015). Genetic risk of neurodegenerative diseases is associated with mild cognitive impairment and conversion to dementia. Alzheimers Dement.

[28] Licher S, Ahmad S, Karamujic-Comic H, Voortman T, Leening MJG, Ikram MA (2019). Genetic predisposition, modifiable-risk-factor profile and long-term dementia risk in the general population. Nat Med.

[29] Sun Y, Yuan S, Chen X, Sun J, Kalla R, Yu L (2023). The contribution of genetic risk and lifestyle factors in the development of adult-onset inflammatory bowel disease: a prospective cohort study. Am J Gastroenterol.

[30] Hu HY, Wu BS, Ou YN, Ma YH, Huang YY, Cheng W (2022). Tea consumption and risk of incident dementia: A prospective cohort study of 377 592 UK Biobank participants. Transl Psychiatry.

[31] Ma LZ, Zhang YR, Li YZ, Ou YN, Yang L, Chen SD (2023). Cataract, cataract surgery, and risk of incident dementia: a prospective cohort study of 300,823 participants. Biol Psychiatry.

[32] Ye J, Wen Y, Sun X, Chu X, Li P, Cheng B (2021). Socioeconomic deprivation index is associated with psychiatric disorders: an observational and genome-wide gene-by-environment interaction analysis in the UK biobank cohort. Biol Psychiatry.

[33] Gregson J, Sharples L, Stone GW, Burman CF, Ohrn F, Pocock S (2019). Nonproportional hazards for time-to-event outcomes in clinical trials: JACC review topic of the week. J Am Coll Cardiol.

[34] Schober P, Vetter TR (2021). Kaplan-Meier Curves, Log-Rank Tests, and Cox Regression for Time-to-Event Data. Anesth Analg.

[35] O’Caoimh R, Sezgin D, O’Donovan MR, Molloy DW, Clegg A, Rockwood K (2021). Prevalence of frailty in 62 countries across the world: a systematic review and meta-analysis of population-level studies. Age Ageing.

[36] Kasajima M, Eggleston K, Kusaka S, Matsui H, Tanaka T, Son BK (2022). Projecting prevalence of frailty and dementia and the economic cost of care in Japan from 2016 to 2043: a microsimulation modelling study. Lancet Public Health.

[37] Dugravot A, Fayosse A, Dumurgier J, Bouillon K, Rayana TB, Schnitzler A (2020). Social inequalities in multimorbidity, frailty, disability, and transitions to mortality: a 24-year follow-up of the Whitehall II cohort study. Lancet Public Health.

[38] Tazzeo C, Rizzuto D, Calderon-Larranaga A, Roso-Llorach A, Marengoni A, Welmer AK (2021). Multimorbidity patterns and risk of frailty in older community-dwelling adults: a population-based cohort study. Age Ageing.

[39] Xu W, Tan L, Wang HF, Jiang T, Tan MS, Tan L (2015). Meta-analysis of modifiable risk factors for Alzheimer’s disease. J Neurol Neurosurg Psychiatry.

[40] Yu JT, Xu W, Tan CC, Andrieu S, Suckling J, Evangelou E (2020). Evidence-based prevention of Alzheimer’s disease: systematic review and meta-analysis of 243 observational prospective studies and 153 randomised controlled trials. J Neurol Neurosurg Psychiatry.

[41] Kuo K, Zhang YR, Chen SD, He XY, Huang SY, Wu BS (2023). Associations of grip strength, walking pace, and the risk of incident dementia: A prospective cohort study of 340212 participants. Alzheimers Dement.

[42] Hackett RA, Davies-Kershaw H, Cadar D, Orrell M, Steptoe A (2018). Walking speed, cognitive function, and dementia risk in the english longitudinal study of ageing. J Am Geriatr Soc.

[43] Esteban-Cornejo I, Ho FK, Petermann-Rocha F, Lyall DM, Martinez-Gomez D, Cabanas-Sanchez V (2022). Handgrip strength and all-cause dementia incidence and mortality: findings from the UK Biobank prospective cohort study. J Cachexia Sarcopenia Muscle.

[44] Zhang Y, Wang J, Zhang Y, Wang L, Wei J (2022). Characteristics scanning of brain structure and function changes in patients with different degrees of Alzheimer’s disease. Contrast Media Mol Imaging.

[45] Sulkava S, Haukka J, Sulkava R, Laatikainen T, Paunio T (2022). Association between psychological distress and incident dementia in a population-based cohort in Finland. JAMA Netw Open.

[46] Islamoska S, Ishtiak-Ahmed K, Hansen AM, Grynderup MB, Mortensen EL, Garde AH (2019). Vital exhaustion and incidence of dementia: results from the copenhagen city heart study. J Alzheimers Dis.

[47] Kivimaki M, Luukkonen R, Batty GD, Ferrie JE, Pentti J, Nyberg ST (2018). Body mass index and risk of dementia: analysis of individual-level data from 1.3 million individuals. Alzheimers Dement.

[48] Qizilbash N, Gregson J, Johnson ME, Pearce N, Douglas I, Wing K (2015). BMI and risk of dementia in two million people over two decades: a retrospective cohort study. Lancet Diabetes Endocrinol.

[49] Tian Q, Studenski SA, An Y, Kuo PL, Schrack JA, Wanigatunga AA (2021). Association of combined slow gait and low activity fragmentation with later onset of cognitive impairment. JAMA Netw Open.

[50] Del Pozo Cruz B, Del Pozo-Cruz J (2021). Associations between activity fragmentation and subjective memory complaints in middle-aged and older adults. Exp Gerontol.

[51] Wanigatunga AA, Di J, Zipunnikov V, Urbanek JK, Kuo PL, Simonsick EM (2019). Association of total daily physical activity and fragmented physical activity with mortality in older adults. JAMA Netw Open.

[52] De la Rosa A, Olaso-Gonzalez G, Arc-Chagnaud C, Millan F, Salvador-Pascual A, Garcia-Lucerga C (2020). Physical exercise in the prevention and treatment of Alzheimer’s disease. J Sport Health Sci.

[53] Theou O, O’Connell MD, King-Kallimanis BL, O’Halloran AM, Rockwood K, Kenny RA (2015). Measuring frailty using self-report and test-based health measures. Age Ageing.

