# Supplementary material for: Physical frailty, genetic predisposition, and incident dementia: a large prospective cohort study
Source: Transl Psychiatry. 2024 May 27;14:212. doi: 10.1038/s41398-024-02927-7 (PMC11130190; doi:10.1038/s41398-024-02927-7)
Supplement: Supplementary file 1 — Supplementary Material [file 41398_2024_2927_MOESM1_ESM.docx]

**Physical frailty, genetic predisposition, and incident dementia: a large prospective cohort study**

Supplementary Material

**Content**

[Supplementary eTable 1. The list of frailty definition in the UK Biobank 3](#_Toc161431821)

[Supplementary eTable 2. List of the ACD, AD, and VaD codes used in this study. 5](#_Toc161431822)

[Supplementary eTable3. The list of long-term morbidities. 7](#_Toc161431823)

[Supplementary eTable 4. Association between dementia polygenic risk score and risk of incident dementia. 10](#_Toc161431824)

[Supplementary eTable 5. Association between physical frailty and incident dementia for subgroups analysis. 11](#_Toc161431825)

[Supplementary eFigure 1. Flowchart of the present study. 13](#_Toc161431826)

[Supplementary eFigure 2. The histogram of frequency distribution of polygenic risk score. 14](#_Toc161431827)

[Supplementary eFigure 3. Kaplan-Meier survival curve for the time to dementia incidence for the classification of physical frailty during follow-up with log-rank test. 15](#_Toc161431828)

[Supplementary eFigure 4. Kaplan-Meier survival curve for the time to dementia incidence for the PRS groups during follow-up with log-rank test. 16](#_Toc161431829)

[Supplementary eFigure 5. Kaplan-Meier survival curve for the time to dementia incidence for the classification of physical frailty and PRS groups during follow-up with log-rank test. 17](#_Toc161431830)

[Reference 18](#_Toc161431831)

| Supplementary eTable 1. The list of frailty definition in the UK Biobank | | |  |
| --- | --- | --- | --- |
| Components | Definition | Field ID | |
| Slow walking speed | Self-reported: “How would you describe your usual walking pace?” Response: slow = 1; other = 0; Do not know/Prefer not to answer = missing data. | 924 | |
| Low physical activity | Self-reported: "In the last 4 weeks did you spend any time doing the following" Response:  Light DIY (eg: pruning, watering the lawn): frequency of once per week or less = 1, more than once a week = 0 None of above = 1 Walking for pleasure (not as a means of transport) = 0 Other exercises (eg: swimming, cycling, keep fit, bowling) = 0 Strenuous sports = 0 Heavy DIY (eg: weeding, lawn mowing, carpentry, digging) = 0 Prefer not to answer (Excluded) | 6164 | |
| Exhaustion | Self-reported: “Over the past two weeks, how often have you felt tired or had little energy?” Response: more than half the days or nearly every day = 1; other = 0; Do not know/Prefer not to answer = missing data. | 2080 | |
| Low grip strength | Measured grip strength expressed in kg by sex- and BMI- adjusted cut-off points.  Cut-off points:  Men  If BMI ≤24.0 kg/m2 & grip strength ≤29 kg  If BMI 24.1 to 26.0 kg/m2 & grip strength ≤30 kg  If BMI 26.1 to 28.0 kg/m2 & grip strength ≤30 kg  If BMI >28.0 kg/m2 & grip strength ≤32 kg  Women If BMI ≤23.0 kg/m2 & grip strength ≤17 kg  If BMI 23.1 to 26.0 kg/m2 & grip strength ≤17.3 kg If BMI 26.1 to 29.0 kg/m2 & grip strength ≤18 kg  If BMI >29.0 kg/m2 & grip strength ≤21 kg If data on BMI or grip strength is not available = missing data. | 46, 47 | |
| Weight loss | Self-reported: “Compared with one year ago, has your weight changed?” Response: yes, lost weight = 1; other = 0; Do not know/Prefer not to answer = missing data. | 2306 | |
| Abbreviations: BMI, body mass index; MET, metabolic equivalent of energy; IPAQ, International Physical Activity Questionnaire. | | | |

| Supplementary eTable 2. List of the ACD, AD, and VaD codes used in this study. | | |
| --- | --- | --- |
|  | Code Types | Codes |
| All-cause dementia | ICD-9 | 290.2, 290.3, 290.4, 291.2, 294.1, 331.0, 331.1, 331.2, 331.5 |
|  | ICD-10 | A81.0, F00, F00.0, F00.1, F00.2, F00.9, F01, F01.0, F01.1, F01.2, F01.3, F01.8, F01.9, F02, F02.0, F02.1, F02.2, F02.3, F02.4, F02.8, F03, F05.1, F10.6, G30, G30.0, G30.1, G30.8, G30.9, G31.0, G31.1, G31.8, I67.3 |
|  | Read V2 | 1461, A411., A4110, E00.., E000., E001., E0010, E0011, E0012, E0013, E001z, E002., E0020, E0021, E002z, E003., E004., E0040, E0041, E0042, E0043, E004z, E012., E02y1, E041., Eu00., Eu000, Eu001, Eu002, Eu00z, Eu01., Eu010, Eu011, Eu012, Eu013, Eu01y, Eu01z, Eu02., Eu020, Eu021, Eu022, Eu023, Eu024, Eu025, Eu02y, Eu02z, Eu041, Eu106, Eu107, F110., F1100, F1101, F111., F112., F116., F118., F11x2, F11x7, F11x9, F11y2, F21y2, Fyu30, 38C13, 3AE3., 3AE4., 3AE5., 3AE6., 66h.., 6AB.., 8BM02, 8BM50, 8BM60, 8BPa., 8CMe0, 8CMG2, 8CMZ., 8CMZ0, 8CMZ1, 8CMZ2, 8CMZ3, 8CSA., 8Hla., 8IAe0, 8IAe2, 9hD.., 9hD0., 9hD1., 9Ou.., 9Ou1.,9Ou2., 9Ou3., 9Ou4., 9Ou5. |
|  | Read | .1461,1461, .E11., .E111, .E112, .E113, .E114, .E115, .E116, .E11Z, .F21Z |
|  | CTV3 | .F371, .G78., A411., A4110, E00.., E000., E001., E0010, E0011, E0012, E0013, E001z, E002., E0020, E0021, E002z, E003., E004., E0040, E0041, E0042, E0043, E004z, E012., E02y1, E041., Eu00., Eu000, Eu001, Eu002, Eu00z, Eu01., Eu010, Eu011, Eu012, Eu013, Eu01y, Eu01z, Eu02., Eu020, Eu021, Eu022, Eu023, Eu024, Eu025, Eu02y, Eu02z, Eu041, F110., F1100, F1101, F111., F112., F116., F118., F11x2, F11x7, F11y2, F21y2, Fyu30, Ub1T6, X002m, X002w, X002x, X002y, X002z, X0030, X0031, X0032, X0033, X0034, X0035, X0036, X0037, X0039, X003A, X003B, X003C, X003D, X003E, X003F, X003G, X003H, X003I, X003J, X003l, X003m, X003P, X003R, X003T, X003V, X003W, X003X, X00R2, X00Rk, Xa0lH, Xa0sC, Xa0sE, Xa1GB, Xa25J, Xa3ez, XaA1S, XabVp, XaE74, XaIKB, XaIKC, XaKyY, XaOfZ, XE17j, XE1aG, XE1Xs, XE1Xu,XE1Z6, .3AE3, .3AE4, .3AE5, .3AE6, .66h., .6AB., .9hD1, .9Ou., .9Ou1, .9Ou2, .9Ou3, .9Ou4, .9Ou5, 3AE3., 3AE4., 3AE5., 3AE6., 66h.., 6AB.., 8BM02, 8BM50, 8BPa., 8CMe0, 8CMG2, 8CMZ., 8CMZ0, 8CMZ1, 8CMZ2, 8CMZ3, 8CSA., 8IAe0, 8IAe2, 9hD1., 9Ou., 9Ou1., 9Ou2., 9Ou3., 9Ou4., 9Ou5., Xa0fZ, XaaBZ, XaaeA, XaaiW, Xabd2, Xabd3, XabEk, XabEl, XabtQ, XacIx, XacIy, XacIz, XacJ0, XacLx, Xacly, Xaclz, XacM2, Xaefu, XaJBQ, XaJBU, XaJBV, XaJBW, XaJBX, XaJPy, XaLFf, XaLFo, XaLFp, XaMFy, XaMG0, XaMGF, XaMGG, XaMGI, XaMGJ, XaMGK, XaMJC, XaYFR, XaYPX, XaZqJ, XaZWz |
| Abbreviations: ACD, All-Cause Dementia; ICD, International Classification of Diseases; Read V2, Read codes version 2; Read CTV3, Read codes version 3. | | |

| Supplementary eTable3. The list of long-term morbidities. | | |
| --- | --- | --- |
|  | Comorbidities | Code |
| 1 | Hypertension | 1065, 1072 |
| 2 | Coronary heart disease | 1074, 1075 |
| 3 | Diabetes | 1607, 1468, 1220, 1222, 1223, 1276 |
| 4 | Stroke/TIA | 1081, 1082, 1086, 1491, 1583 |
| 5 | Atrail fibrillation | 1471 |
| 6 | Heart failure | 1079, 1588, 1076 |
| 7 | Peripheral vascular disease | 1067, 1087 |
| 8 | COPD | 1112, 1113, 1472 |
| 9 | Asthma | 1111 |
| 10 | Bronchiectasis | 1114 |
| 11 | Cancer | - |
| 12 | Dyspepsia | 1138, 1139, 1142, 1143, 1457, 1510, 1474, 1442 |
| 13 | Diverticular disease | 1458 |
| 14 | Irritable bowel syndrome | 1154 |
| 15 | Chronic liver disease | 1141, 1157, 1158, 1506 |
| 16 | Inflammatory bowel disease | 1461, 1462, 1463 |
| 17 | Constipation | 1599 |
| 18 | Viral hepatitis | 1579, 1580, 1581 |
| 19 | Depression | 1286, 1531 |
| 20 | Anxiety | 1288, 1469, 1615, 1614, 1616, 1243 |
| 21 | Schizophrenia/Bipolar affective disorder | 1289, 1291 |
| 22 | Connective tissue diseases | 1322, 1381, 1373, 1382, 1383, 1384, 1464, 1477, 1480, 1481, 1377 |
| 23 | Painful conditions | 1534, 1537, 1436, 1476, 1540, 1541, 1542, 1538, 1573, 1532, 1312, 1311, 1294, 1465, 1466, 1478, 1523, 1533, 1257 |
| 24 | Osteoporosis | 1309 |
| 25 | Thyroid disorders | 1224, 1225, 1226, 1522, 1610, 1428 |
| 26 | Alcohol problems | 1408, 1604 |
| 27 | Chronic kidney disease | 1427, 1607, 1192, 1193, 1194, 1519, 1520 |
| 28 | Prostate disorders | 1207, 1396, 1516 |
| 29 | Glaucoma | 1277 |
| 30 | Epilepsy | 1264 |
| 31 | Dementia | 1263* |
| 32 | Psoriasis or eczema | 1452, 1453 |
| 33 | Migraine | 1265 |
| 34 | Chronic sinusitis | 1416 |
| 35 | Anorexia or bulimia | 1470 |
| 36 | Parkinson’s disease | 1262 |
| 37 | Multiple sclerosis | 1261 |
| 38 | Chronic fatigue syndrome | 1482 |
| 39 | Endometriosis | 1402 |
| 40 | Meniere disease | 1421 |
| 41 | Pernicious anaemia | 1331 |
| 42 | Polycystic ovaries | 1350 |
| Self-reported lifetime physician’s diagnoses were documented through nurse-led interviews (UK Biobank Field ID: 20002), with the exception of cancer diagnoses, which were reported using a touchscreen questionnaire. The classification of diseases was based on Barnett et al (2012)^1^.  *In our study, the self-report dementia (Code: 1263) diagnosis were not included.  Further details were shown in https://biobank.ndph.ox.ac.uk/ukb/field.cgi?id=20002 | | |

| Supplementary eTable 4. Association between dementia polygenic risk score and risk of incident dementia. | | | | |
| --- | --- | --- | --- | --- |
| PRS Group | Cases/person-years | Incident cases per 100,000 person-years | HR (95% CI) | *P* |
| Low | 529/477004 | 110.9 | 1 (Reference) |  |
| Intermediate | 1917/1431327 | 133.9 | 1.210 (1.099-1.332) | <0.001 |
| High | 907/476846 | 190.2 | 1.720 (1.545-1.915) | <0.001 |
| Model adjusted for genotyping array and the first 10 principal components of ancestry. Abbreviations: HR: Hazard ratio; CI: confidence interval; PRS, polygenic risk score. | | | | |

| Supplementary eTable 5. Association between physical frailty and incident dementia for subgroups analysis. | | | | | | | | |
| --- | --- | --- | --- | --- | --- | --- | --- | --- |
|  | | Frailty phenotype | | | | | | |
|  |  | Non-frailty | Prefrailty | |  | Frailty | |  |
|  | N | HR (95%CI) | HR (95%CI) | *P* | *P* for interaction | HR (95%CI) | *P* | *P* for interaction |
| Age | | | | | | | | |
| Age<60 | 145,212 | 1 (reference) | 2.122 (1.680-2.680) | <0.001 | <0.001 | 3.887 (2.723-5.550) | <0.001 | <0.001 |
| Age≥60 | 128,982 | 1 (reference) | 1.325 (1.223-1.436) | <0.001 |  | 2.142 (1.868-2.456) | <0.001 |  |
| Sex | | | | | | | | |
| Female | 146,574 | 1 (reference) | 1.410 (1.254-1.585) | <0.001 | 0.545 | 2.055 (1.705-2.477) | <0.001 | 0.163 |
| Male | 127,630 | 1 (reference) | 1.378 (1.247-1.522) | <0.001 |  | 2.568 (2.158-3.055) | <0.001 |  |
| Townsend deprivation index | | | | | | | | |
| Lower level (below median) | 136,946 | 1 (reference) | 1.356 (1.217-1.511) | <0.001 | 0.75 | 2.183 (1.858-2.565) | <0.001 | 0.864 |
| Higher level (above median) | 136,919 | 1 (reference) | 1.438 (1.293-1.599) | <0.001 |  | 2.433 (1.964-3.013) | <0.001 |  |
| Number of morbidities | | | | | | | | |
| 0 | 95,817 | 1 (reference) | 1.603 (1.358-1.891) | <0.001 | 0.037 | 3.248 (1.994-5.289) | <0.001 | 0.022 |
| 1 | 87,984 | 1 (reference) | 1.436 (1.255-1.643) | <0.001 |  | 2.806 (2.108-3.734) | <0.001 |  |
| 2 or more | 90,393 | 1 (reference) | 1.276 (1.144-1.424) | <0.001 |  | 2.022 (1.734-2.357) | <0.001 |  |
| Model adjusted for age, sex, Townsend deprivation index, assessment centers, alcohol consumption, smoking status, BMI, the number of long-term morbidities, polygenic risk score, genotyping array, and the first 40 principal components of ancestry. | | | | | | | | |

# Supplementary eFigure 1. Flowchart of the present study.


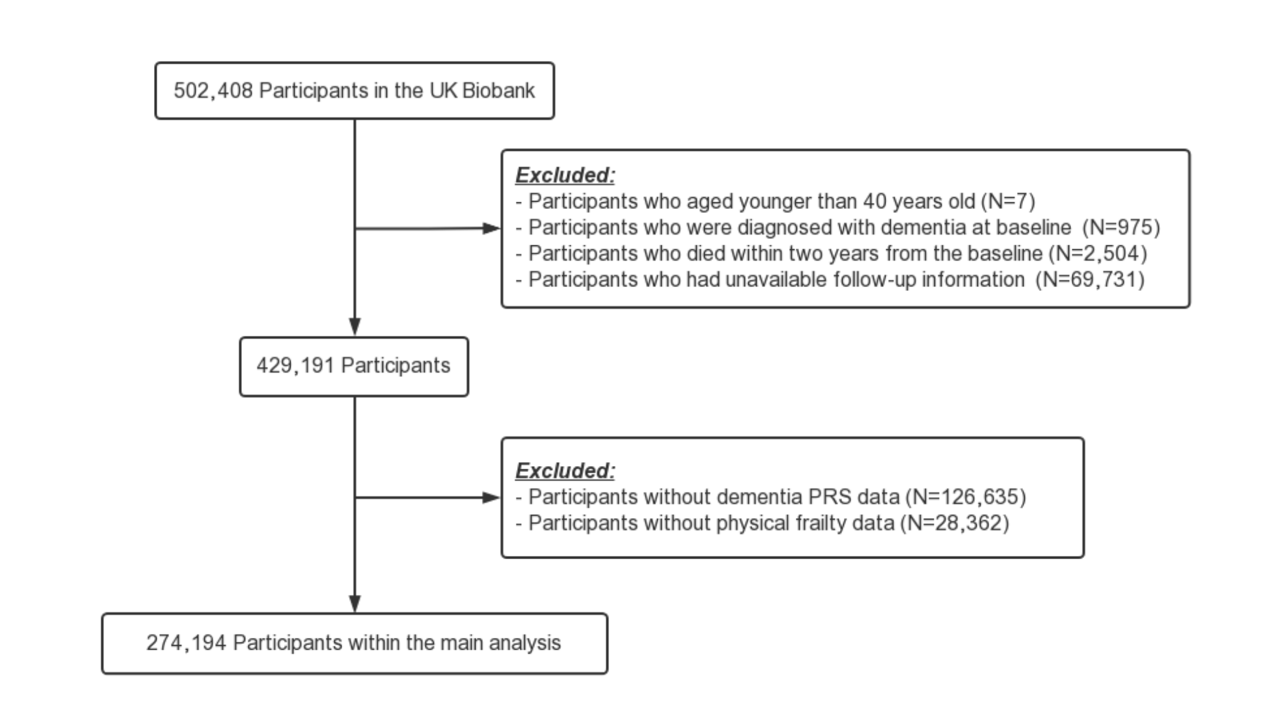


Supplementary eFigure 2. The histogram of frequency distribution of polygenic risk score.


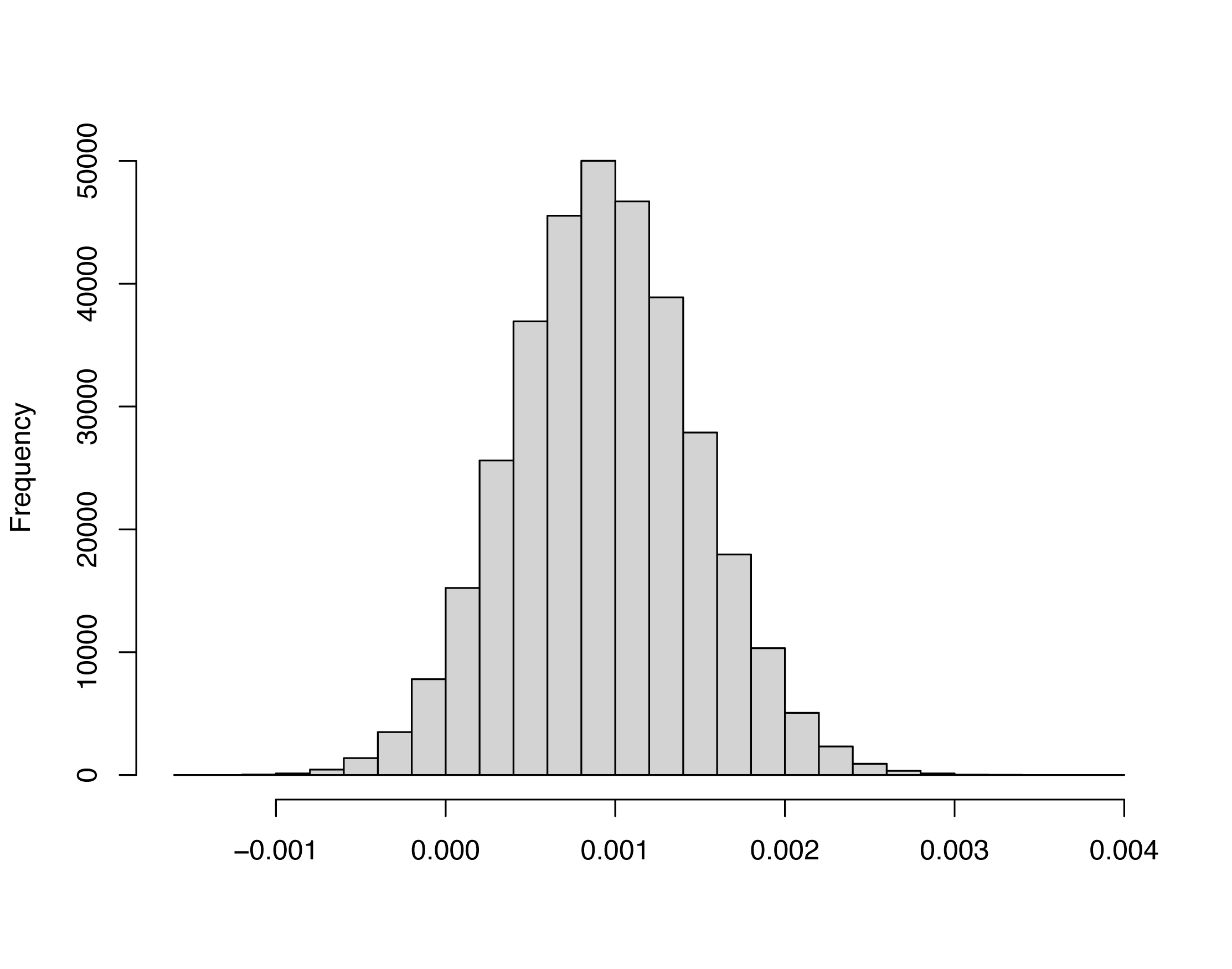


Supplementary eFigure 3. Kaplan-Meier survival curve for the time to dementia incidence for the classification of physical frailty during follow-up with log-rank test.


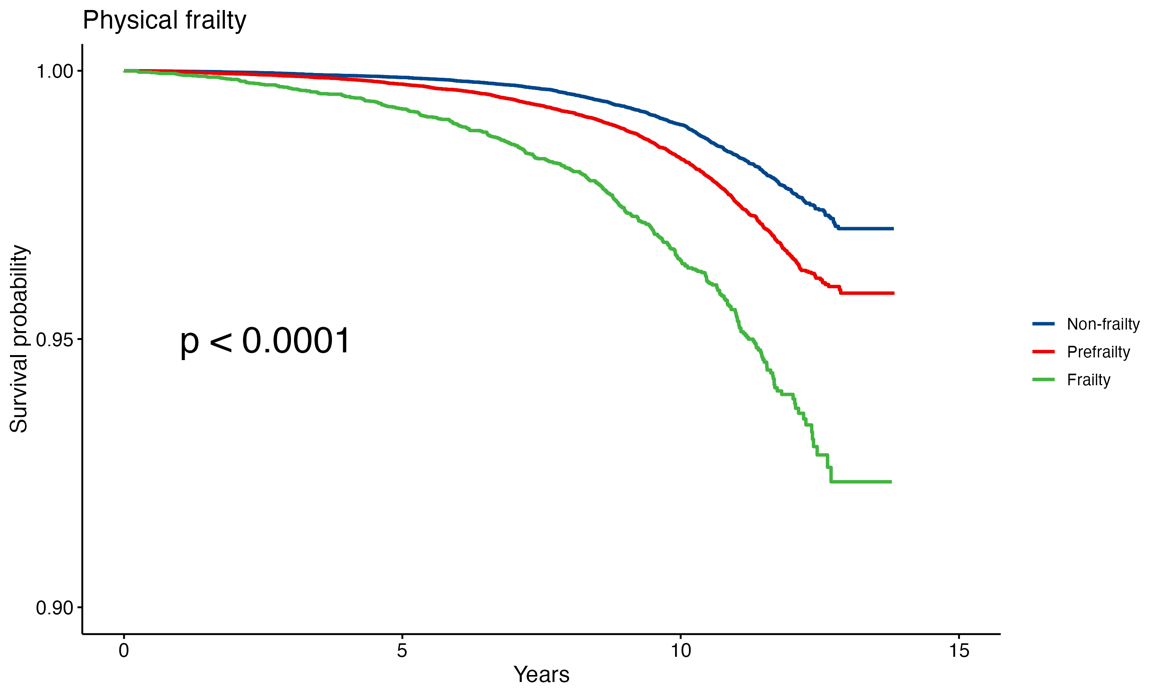


Supplementary eFigure 4. Kaplan-Meier survival curve for the time to dementia incidence for the PRS groups during follow-up with log-rank test.


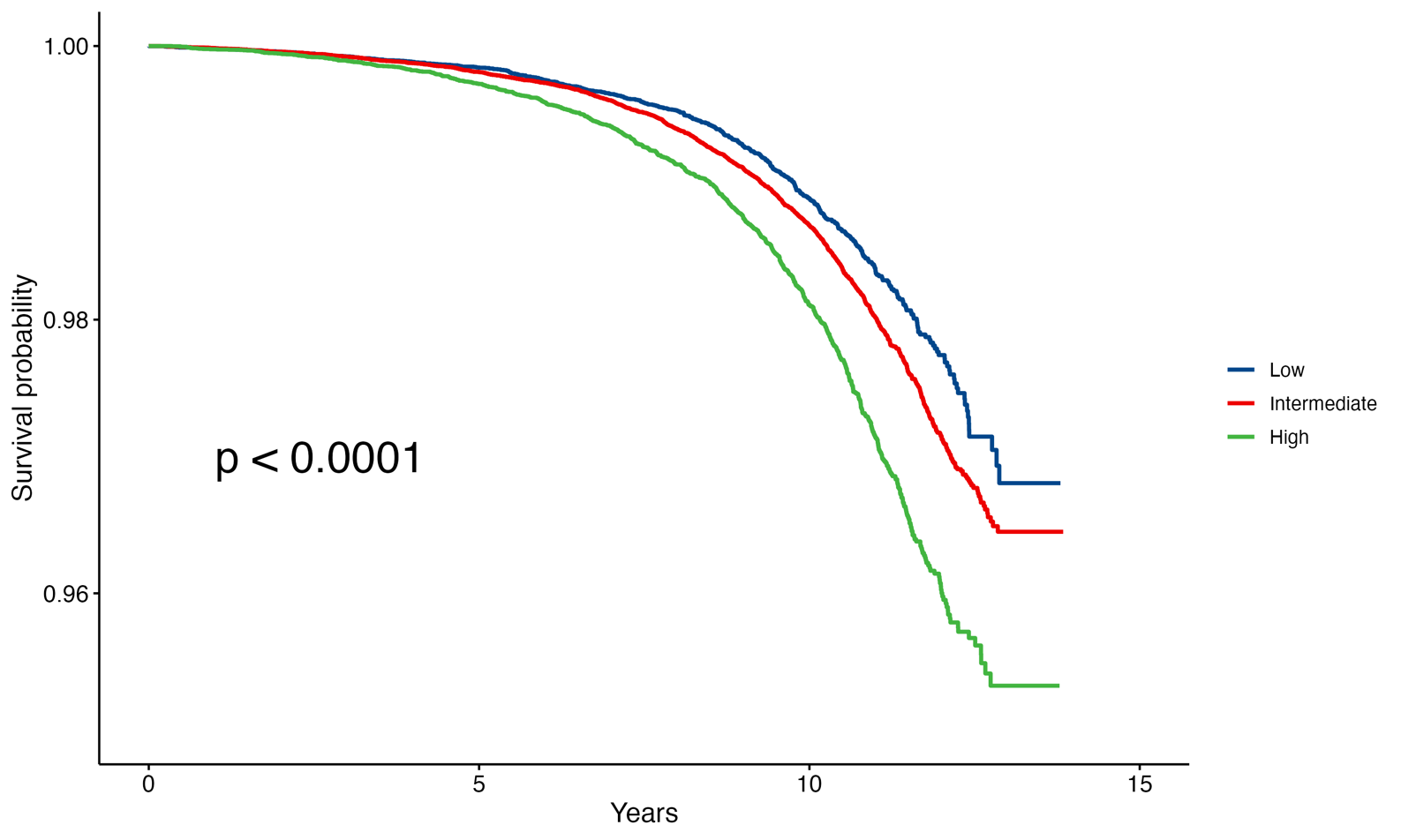


Supplementary eFigure 5. Kaplan-Meier survival curve for the time to dementia incidence for the classification of physical frailty and PRS groups during follow-up with log-rank test.


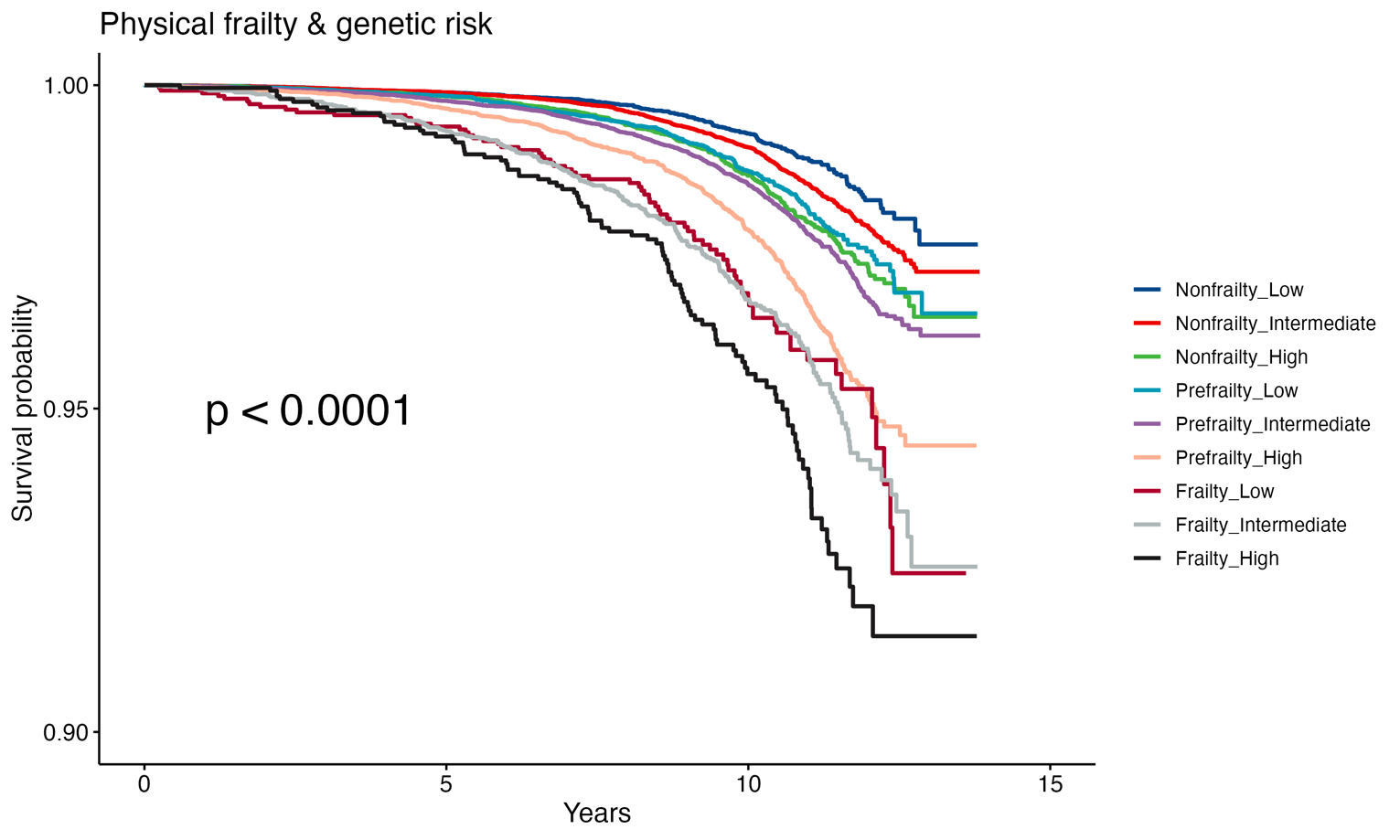


Reference

1. Barnett K, Mercer SW, Norbury M, Watt G, Wyke S, Guthrie B. Epidemiology of multimorbidity and implications for health care, research, and medical education: a cross-sectional study. *Lancet*. Jul 7 2012;380(9836):37-43. doi:10.1016/S0140-6736(12)60240-2
